# Supplementary material for: Resolving the full spectrum of human genome variation using Linked-Reads
Source: Genome Res. 2019 Apr;29(4):635–45. doi: 10.1101/gr.234443.118 (PMC6442396; doi:10.1101/gr.234443.118)
Supplement: Supplemental Material [file supp_gr.234443.118_Supplemental_longranger-2.2.zip › longranger-2.2.tar.bz2!/lr2.2-public/lib/stan/cmdstan/stan/doc/api/html/agrad_2rev_2functions_2is__uninitialized_8hpp_source.html]

Stan: src/stan/agrad/rev/functions/is\_uninitialized.hpp Source File


|  |  |
| --- | --- |
|  | Stan  2.5.0  probability, sampling & optimization |


- Main Page
- Related Pages
- Namespaces
- Classes
- Files

- File List
- File Members

All Classes Namespaces Files Functions Variables Typedefs Enumerations Enumerator Friends Macros Pages

- src
- stan
- agrad
- rev
- functions

is\_uninitialized.hpp

Go to the documentation of this file.

1 #ifndef STAN\_\_AGRAD\_\_REV\_\_FUNCTIONS\_\_IS\_UNINITIALIZED\_HPP

2 #define STAN\_\_AGRAD\_\_REV\_\_FUNCTIONS\_\_IS\_UNINITIALIZED\_HPP

3

4 #include <stan/agrad/rev/var.hpp>

5 #include <stan/agrad/rev/vari.hpp>

6 #include <stan/agrad/rev/operators/operator\_unary\_negative.hpp>

7 #include <stan/math/functions/is\_uninitialized.hpp>

8

9 namespace stan {

10

11  namespace agrad {

12

25  inline bool is\_uninitialized(var x) {

26  return x.is\_uninitialized();

27  }

28

29  }

30 }

31 #endif

vari.hpp

var.hpp

stan::agrad::var::is\_uninitialized

bool is\_uninitialized()

Return true if this variable has been declared, but not been defined.

**Definition:** var.hpp:51

stan::agrad::var

Independent (input) and dependent (output) variables for gradients.

**Definition:** var.hpp:27

stan::agrad::is\_uninitialized

bool is\_uninitialized(var x)

Returns true if the specified variable is uninitialized.

**Definition:** is\_uninitialized.hpp:25

is\_uninitialized.hpp

operator\_unary\_negative.hpp


---

[ Stan Home Page ]

*© 2011–2014,
Stan Development Team.*
